# Supplementary material for: Symptom Severity and Health-Related Quality of Life in Patients with Atrial Fibrillation: Findings from the Observational ARENA Study
Source: J Clin Med. 2022 Feb 21;11(4):1140. doi: 10.3390/jcm11041140 (PMC8877113; doi:10.3390/jcm11041140)
Supplement: Supplementary file 1 [file jcm-11-01140-s001.zip › jcm-1561521-supplementary.pdf]

Table S1. Parameter estimates of a multiple regression model predicting EHRA scores concurrently from all significant predictors in the models reported in the main manuscript (compare Table 3 and Table 4).

		EHRA class 2a or 2b					EHRA class 3 or 4				
	**Predictor**	**Coef.**	**95% CI**	*z*	**p**	**OR**	**Coef.**	**95% CI**	*z*	**p**	**OR**
Confounders	(Intercept)	1.684	0.548; 2.821	2.904	0.004	5.390	0.735	-0.732; 2.203	0.982	0.326	2.086
	Age	-0.037	-0.053; -0.022	-4.709	<0.001	0.963	-0.049	-0.069; -0.029	-4.773	<0.001	0.952
	Sex (female)	0.435	0.143; 0.726	2.921	0.003	1.544	1.245	0.869; 1.621	6.495	<0.001	3.473
	AF Persistent	0.192	-0.128; 0.512	1.175	0.240	1.211	0.380	-0.042; 0.801	1.767	0.077	1.462
	AF Permanent	-0.464	-0.843; -0.085	-2.399	0.016	0.629	-0.315	-0.850; 0.220	-1.155	0.248	0.730
H2	CAD	0.037	-0.285; 0.359	0.223	0.823	1.037	0.517	0.068; 0.966	2.258	0.024	1.678
	Cardioversion	0.358	0.069; 0.647	2.428	0.015	1.431	0.273	-0.113; 0.660	1.387	0.165	1.314
H3	Sleep disturbance	0.230	0.067; 0.393	2.769	0.006	1.259	0.194	-0.023; 0.412	1.753	0.080	1.215
	Stress Work	0.033	-0.152; 0.218	0.353	0.724	1.034	0.276	0.046; 0.506	2.351	0.019	1.318
	Stress Financial	-0.396	-0.660; -0.132	-2.939	0.003	0.673	-0.515	-0.871; -0.160	-2.840	0.005	0.597
	Stress Noise	0.353	0.127; 0.579	3.066	0.002	1.424	0.170	-0.134; 0.474	1.098	0.272	1.186
	CAQ-2	0.184	0.106; 0.263	4.594	<0.001	1.202	0.194	0.090; 0.299	3.650	<0.001	1.215

Note. AF = atrial fibrillation; CAD = Coronary artery disease; CAQ-2 = Cardiac Anxiety Questionnaire 2-item screener; H2 = Hypothesis 2; H3 = Hypothesis 3.

Table S2. Parameter estimates of a multiple regression model predicting EQ-5D-5L scores concurrently from all significant predictors in the models reported in the main manuscript (compare Table 5 and Table 6).

Hypothesis	Predictor	Estimate	95% CI	β	t-value	p
Confounders	(Intercept)	1.310	1.225; 1.394	0.158	30.484	<0.001
	Age	-0.004	-0.005; -0.003	-0.160	-6.248	<0.001
	Sex (female)	-0.019	-0.044; 0.006	-0.077	-1.496	0.135
	Persistent AF	0.006	-0.022; 0.033	0.023	0.407	0.684
	Permanent AF	-0.054	-0.087; -0.020	-0.218	-3.126	0.002
H2	CAD	-0.024	-0.053; 0.004	-0.099	-1.685	0.092
	DM	-0.033	-0.061; -0.005	-0.133	-2.274	0.023
H3	Sleep disturbance	-0.031	-0.045; -0.017	-0.111	-4.249	<0.001
	Stress Financial	-0.052	-0.076; -0.029	-0.116	-4.341	<0.001
	GAD-2	-0.058	-0.068; -0.048	-0.320	-11.027	<0.001
	CAQ-2	-0.021	-0.028; -0.014	-0.155	-5.801	<0.001

Note. AF = atrial fibrillation; CAD = Coronary artery disease; CAQ-2 = Cardiac Anxiety Questionnaire 2-item screener;DM = Diabetes mellitus; GAD-2 = Generalized Anxiety Disorder 2-item screener; H2 = Hypothesis 2; H3 = Hypothesis 3.
